# Assessing metastasis risk after pre-operative anti-angiogenic therapy

**DOI:** 10.15252/emmm.201404640

**Published:** 2014-11-13

**Authors:** Daniela Biziato, Michele De Palma

**Affiliations:** 1 The Swiss Institute for Experimental Cancer Research (ISREC), School of Life Sciences, École Polytechnique Fédérale de Lausanne (EPFL)Lausanne, SwitzerlandE-mail: michele.depalma@epfl.chDOI 10.15252/emmm.201404640 | Published online 13 November 2014

## Abstract

Anti-angiogenic drugs are approved for the treatment of several cancer types, generally in the inoperable locally advanced or metastatic setting and in combination with other anti-cancer agents. Recent clinical studies also suggest that anti-angiogenic drugs can be useful in the pre-operative (neoadjuvant) setting, by facilitating the shrinkage of the primary tumour and its surgical resection. However, the effects of neoadjuvant anti-angiogenic therapy on the ability of tumours to form distant metastases are unclear. In this issue of *EMBO Molecular Medicine*, Ebos *et al* (2014) present carefully performed pre-clinical studies in mice that analyse the effects of pre-operative anti-angiogenic therapy on tumour metastasis and survival.

See also: **JML Ebos *et al*** (December 2014)

Several angiogenesis inhibitors are currently employed for the treatment of advanced and/or metastatic cancer, often in combination with other anti-cancer agents. Bevacizumab, a monoclonal antibody that neutralizes the vascular-endothelial growth factor (VEGF)-A, was the first to receive approval in 2004. Among other indications, it is now used in combination with chemotherapy for the first-line treatment of metastatic colorectal and non-small cell lung cancer and, as a single agent, for recurrent glioblastoma. Other approved anti-angiogenic agents include multi-kinase inhibitors such as sunitinib and sorafenib, which target the VEGF receptors (VEGFRs) and other kinases with pro-angiogenic and pro-proliferative functions (Sennino & McDonald, 2012). Compared to the previous standard of care, treatments based on angiogenesis inhibitors provide benefits in terms of objective response, which translate into frequent but short-lived improvements in progression-free and overall survival. The lack of predictive biomarkers of response, which may help identify patients who are more likely to benefit, and the emergence of resistance to therapy are believed to limit the clinical efficacy of anti-angiogenic drugs in late-stage cancer (Bergers & Hanahan, 2008; Sennino & McDonald, 2012).

Angiogenesis inhibitors are not yet approved for the pre-operative (neoadjuvant) treatment of resectable cancer. While these drugs may promote the shrinkage and, therefore, facilitate the surgical resection of the tumour, concerns also exist that they might concomitantly increase its propensity to form distant metastasis. Indeed, studies in mice have shown that tumour blood vessel pruning may stimulate cancer cells to acquire pro-invasive and metastatic traits, a threatening form of tumour adaptation to the hypoxic microenvironment (Sennino & McDonald, 2012). Although these experimental findings suggest that the immediate benefits of pre-operative anti-angiogenic therapy might be countered in the long term by a heightened metastasis risk, a constellation of parameters (e.g. drug mode of action, dose and scheduling; combination with other anti-cancer drugs; the cancer type/model) may affect the metastatic behaviour of the tumour on-treatment.

Ebos *et al* (2014) compared the effects of different classes and dosage regimens of anti-angiogenic drugs (including kinase inhibitors and VEGFA/VEGFR blocking antibodies) and a vascular-disrupting agent (OXi4503), alone or in combination with low-dose cytotoxic chemotherapy (cyclophosphamide/5-fluorouracil), on the metastatic spread of tumours treated pre-operatively in mice. They employed bioluminescent human cancer cell lines (representing breast, melanoma and kidney cancer) that spontaneously metastasize to several organs when implanted orthotopically, that is in their native site, in immunodeficient mice. After surgical removal of the primary tumour, the progressive growth of the metastases was monitored in live mice by measuring bioluminescence (Fig1A). Termination criteria were also established to determine mouse survival.

**Figure 1:**
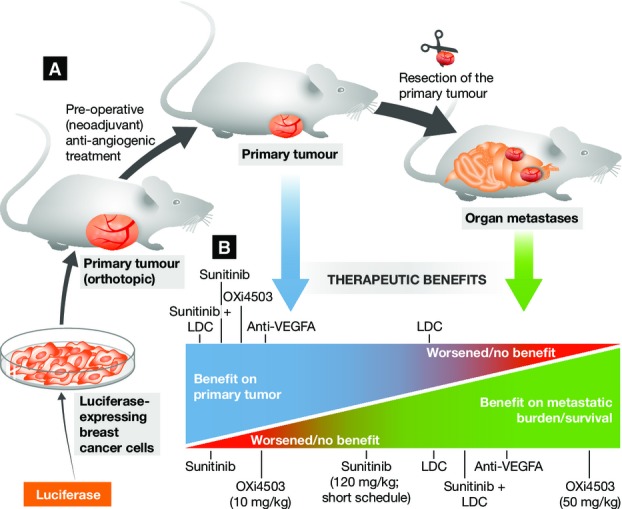
Testing neoadjuvant anti-angiogenic therapies in an excisional breast cancer model (A) A human breast cancer cell line with metastatic capability is genetically modified with a luciferase construct to allow *in vivo* tracing. Primary tumour growth is initiated by orthotopically transplanting the cancer cells in the mammary fat pad of severe combined immunodeficient (SCID) mice. Established tumours are then treated with specific drug combinations, including anti-angiogenic agents. The primary tumours are removed and the subsequent formation of metastases is monitored by measuring luciferase activity. (B) The therapeutic benefits of the distinct drugs—alone or in combination, and at different dosage regimens—on the primary tumours and post-surgical metastases are shown (for details on dosage regimens and quantitative data, refer to Ebos *et al* (2014)). Note that Ebos *et al* (2014) investigated several tumour models; for the sake of simplicity, only the breast cancer model is exemplified in the figure. LDC, low-dose chemotherapy (cyclophosphamide plus 5-fluorouracil).

The authors observed tumour-type and drug-dependent effects of neoadjuvant anti-angiogenic therapy on the development of metastasis post-surgery (Fig1B). For example, high-dose (60 mg/kg) sunitinib—a broad-spectrum kinase inhibitor that primarily blocks the VEGFRs and platelet-derived growth factor receptors (PDGFRs)—had variable growth-inhibitory effects on the different primary tumour models tested, but consistently exacerbated post-surgery metastatic growth and worsened survival. These findings are in agreement with previous studies that documented pro-metastatic effects of high-dose sunitinib in non-surgical tumour models (Ebos *et al*, 2009; Pàez-Ribes *et al*, 2009; Chung *et al*, 2012). On the other hand, all antibody-based VEGFA-pathway inhibitors and high-dose (50 mg/kg) OXi4503 had beneficial effects on both the primary tumours and post-surgical metastases. Interestingly, the pro-metastatic effects of sunitinib could be attenuated in a breast cancer model by adopting a “condensed” drug schedule, in which a further higher dose (120 mg/kg) was administered for a shorter time before surgery. Together, these findings strongly suggest that the pro- versus anti-metastatic activities of angiogenesis inhibitors are drug-class and dose dependent.

The aforementioned observations are certainly relevant and timely as ongoing clinical trials evaluate the benefits of neoadjuvant angiogenesis inhibitors in several cancer types. Although survival data are not yet available, two large randomized trials documented a significant increase in the rate of pathological complete response in breast cancer after neoadjuvant bevacizumab plus chemotherapy (Vasudev & Reynolds, 2014). Furthermore, neoadjuvant sunitinib is being investigated in breast cancer (NCT00656669) and metastatic renal cell carcinoma (NCT01-099423). The addition of bevacizumab to neoadjuvant chemotherapy, however, increased the risk of surgical complications in patients undergoing breast-conserving surgery or repeated surgical procedures (Gerber *et al*, 2014). Because the half-life of bevacizumab is about 2–3 weeks, performing surgery at least 6–8 weeks after the last bevacizumab infusion should significantly reduce the occurrence of such complications.

The findings of Ebos *et al* (2014) also raise important questions, which should be addressed in order to better appreciate the clinical relevance and transferability of their findings. For example, how do the dosage regimens described by the authors compare to those employed in patients? Daily doses of sunitinib in the range of 60–120 mg/kg are markedly higher than those administered to cancer patients, so it is unclear whether the reported dose-dependent effects on metastatic growth in mice would be applicable to the clinical setting. Moreover, it would be of interest to see whether both the histo-pathological responses in the primary tumours and the systemic host responses induced by sunitinib differ between the “standard” and “condensed” regimens. Sunitinib may alter tumour growth and metastatic progression through several mechanisms. Besides pruning intratumoural blood vessels by inhibiting endothelial cell proliferation (via VEGFR2 inhibition) and depleting pericytes (via PDGFR inhibition), sunitinib may have direct inhibitory effects on cancer cells (e.g. via STAT3 inhibition) as well as broader effects on a variety of host (non-malignant) cells (Xin *et al*, 2009). For example, it can prevent the activation of the colony-stimulating factor-1 receptor (CSF1R), which conveys essential pro-survival signalling to monocytes and macrophages (Kitagawa *et al*, 2012). These cells have important vascular-modulatory functions and also appear to facilitate the establishment of metastasis by acting at different steps during the metastatic cascade (Qian & Pollard, 2010; Mazzieri *et al*, 2011). Because sunitinib may inhibit different kinases dose dependently and with variable potency, it is tempting to speculate that—at the highest doses tested in mice—it may have reversed its pro-metastatic activity by impairing STAT3-mediated survival of early-disseminated cancer cells, or by depleting metastasis-promoting, CSF1R-dependent inflammatory monocytes.

Importantly, Ebos *et al* (2014) found that low-dose chemotherapy (LDC) could help improve the performance of neoadjuvant sunitinib treatment by extending post-surgical survival in a breast cancer model. Although these findings need further validation, they suggest that the complementary actions of LDC and sunitinib on primary and metastatic tumours can synergize to favour both primary tumour responses and outcome. Indeed, whereas LDC has negligible effects on primary tumour growth while improving post-surgery survival, sunitinib has marked effects on the primary tumour but promotes post-surgical metastatic dissemination. Therefore, drugs that can prevent the dissemination and survival of cancer cells may be combined with multi-kinase angiogenesis inhibitors to improve their safety. In this regard, inhibition of angiopoietin-2 (a pro-angiogenic growth factor that activates the TIE2 receptor) is increasingly recognized as a dual angio-inhibitory and anti-metastatic strategy (Mazzieri *et al*, 2011; Rigamonti *et al*, 2014) that might alleviate the risk of increased metastasis associated with the use of more potent angiogenesis inhibitors.

The majority of the experimental trials reported by Ebos *et al* (2014) were conducted in immunodeficient mice, which lack an intact immune system. As a consequence, the potentially important role played by adaptive immune cells, such as T and B lymphocytes, in the regulation of tumour responses to anti-cancer therapies needs to be studied more thoroughly in immunocompetent mice. Regardless of the current limitations, Ebos *et al* (2014) convincingly show that, at least in mice, primary tumour responses to neoadjuvant anti-angiogenic therapy do not necessarily predict post-surgical disease recurrence and survival. Hopefully, the results of the ongoing and future clinical studies will provide an answer to the most important question of all: does neoadjuvant anti-angiogenic therapy increase the survival of cancer patients?
